# Bilateral Renal Infarction Associated With Left Renal Artery Thrombosis and Predominant Left Renal Infarction: A Case Report

**DOI:** 10.7759/cureus.115411

**Published:** 2026-08-29

**Authors:** Saifatullah Khan, Muftah Othman, Zishan Nasir, Zafar Iqbal, Syed Hidayat Ali

**Affiliations:** 1 Medicine, Hamad Medical Corporation, Doha, QAT; 2 Nephrology, Hamad Medical Corporation, Doha, QAT; 3 Medicine/Nephrology, Hamad General Hospital, Doha, QAT

**Keywords:** acute kidney injury, anticoagulants use, renal artery, renal artery thrombosis., tuberculosis

## Abstract

Although uncommon, bilateral renal infarction and renal artery thrombosis should be considered a potential cause of acute kidney injury. Its nonspecific presentation frequently contributes to delayed diagnosis, thereby compromising timely intervention and increasing the risk of irreversible injury. While embolic and thrombotic mechanisms are the predominant etiologies, idiopathic renal artery thrombosis is rare.

We report the case of a 42-year-old man who presented with sudden-onset left flank pain radiating to the left upper quadrant, associated with nausea and abdominal discomfort. He had a history of tuberculosis diagnosed seven months earlier and had completed a six-month course of anti-tuberculous therapy with isoniazid, rifampin, pyrazinamide, and ethambutol. He had no history of cardiovascular disease, thrombophilia, or autoimmune disorders. Laboratory results revealed a mild elevation in serum creatinine (1.5 mg/dL), with no prior baseline for comparison. Urinalysis was unremarkable. Unexplained flank pain in association with elevated serum creatinine and lactate dehydrogenase raised clinical suspicion for renal vascular pathology. Contrast-enhanced computed tomography of the abdomen and pelvis revealed extensive thrombosis involving the left renal artery, resulting in total left kidney infarction. In addition, a small segmental infarction was seen in the mid pole of the right kidney posteriorly; however, the majority of the right kidney parenchyma is intact and enhancing. The right main renal artery was patent. A vascular surgeon was consulted, who, because of the delayed presentation, recommended conservative management rather than surgical intervention. At the time of presentation, the patient was initially treated with systemic therapeutic anticoagulation. Following clinical stabilization, anticoagulation was transitioned to oral rivaroxaban for continued anticoagulant therapy. Renal function and clinical symptoms progressively improved.

## Introduction

Complete or partial occlusion of the main renal artery or its segmental branches may result in renal ischemic injury and, if not promptly and adequately treated, can progress to ischemic nephropathy. Although renal infarction is a rare and often overlooked cause of renal failure [1], it remains an important potential cause of renal failure. Most commonly, it occurs secondary to thromboembolic events originating from the heart or aorta. In-situ thrombosis of the renal artery is relatively rare. The most common causes of in-situ renal artery thrombosis are blunt abdominal trauma and atherosclerotic disease affecting the renal artery [2]. Renal artery thrombosis has been reported in association with conditions such as polycythemia vera [3], pregnancy, hypercoagulable states, renal transplantation [4], nephrotic syndrome, systemic lupus erythematosus [5], and infective endocarditis [6]. However, spontaneous renal artery thrombosis occurring in the absence of any identifiable underlying cause remains exceedingly rare. Spontaneous renal artery thrombosis in the absence of an identifiable cause or risk factor is exceedingly rare. The clinical presentation of renal infarction is often nonspecific and may overlap with more common conditions, such as renal calculi and pyelonephritis, which can contribute to delays in diagnosis. Its nonspecific presentation may lead to delayed or missed diagnosis, potentially resulting in irreversible loss of renal function. Early recognition of this condition is essential to facilitate prompt initiation of revascularization therapy and optimize renal outcomes [7]. With the advent of contrast-enhanced computed tomography (CT) imaging, the detection of renal infarction has become more feasible and accurate. Early recognition of renal infarction facilitates timely initiation of revascularization therapy, which may improve renal outcomes and preserve renal function. We present a rare case of bilateral renal infarction associated with left renal artery thrombosis causing acute kidney injury in a patient with a recent history of tuberculosis who had completed a six-month course of antituberculous therapy. The patient had a favorable outcome following anticoagulation therapy.

## Case presentation

A 42-year-old man presented to the emergency department with moderate left flank pain that had started 3 days prior to presentation. The pain progressively increased in severity over the preceding three to four hours and became severe on the day of admission. It was associated with nausea and one episode of vomiting. He denied urinary urgency or frequency. His medical history was significant for tuberculosis, for which he had previously received isoniazid, rifampin, pyrazinamide, and ethambutol. He also had a four-pack-year smoking history. On initial examination, the patient was alert and oriented, with a blood pressure of 140/75 mmHg. Abdominal examination revealed left and right upper quadrant tenderness corresponding to the patient's presenting pain, with no abdominal distension, guarding, or rebound tenderness. There was no palpable abdominal mass or organomegaly. A peripheral vascular examination revealed palpable peripheral pulses bilaterally, with no clinical evidence of limb ischemia, peripheral edema, or significant asymmetry. The extremities were warm and well perfused. There were no clinical signs of venous thrombosis. Laboratory investigations revealed an elevated serum creatinine level of 1.5 mg/dL. Urine output was 1500 mL/24 hours, with no evidence of oliguria. The patient was admitted and subsequently underwent initial non-contrast CT of the abdomen and pelvis, performed using a renal stone protocol, which did not demonstrate an obstructing calculus or another clear structural cause for the patient's severe flank pain and acute kidney injury. In view of the persistent unexplained flank pain and markedly elevated serum lactate dehydrogenase, renal vascular pathology was subsequently suspected, and contrast-enhanced CT angiography was performed approximately one hour later. The contrast-enhanced CT demonstrated non-opacification of the left renal artery, consistent with acute arterial thrombosis. Correspondingly, the left kidney showed diffuse and marked reduction/absence of parenchymal enhancement, compatible with extensive renal ischemia and infarction. The right kidney demonstrated a focal posterior segmental wedge-shaped area of reduced enhancement, consistent with a segmental renal infarction; however, the majority of the right kidney parenchyma is intact and enhancing. The right main renal artery is patent. These findings, in combination with the absence of an obstructive etiology, supported the diagnosis of bilateral renal infarction with extensive left renal infarction due to left renal artery thrombosis and focal right renal infarction (Figure 1). The case was discussed with the vascular surgery team, who concluded that there was no indication for surgical or endovascular intervention at that time. Conservative management with continued anticoagulation was therefore recommended.

**Figure 1:**
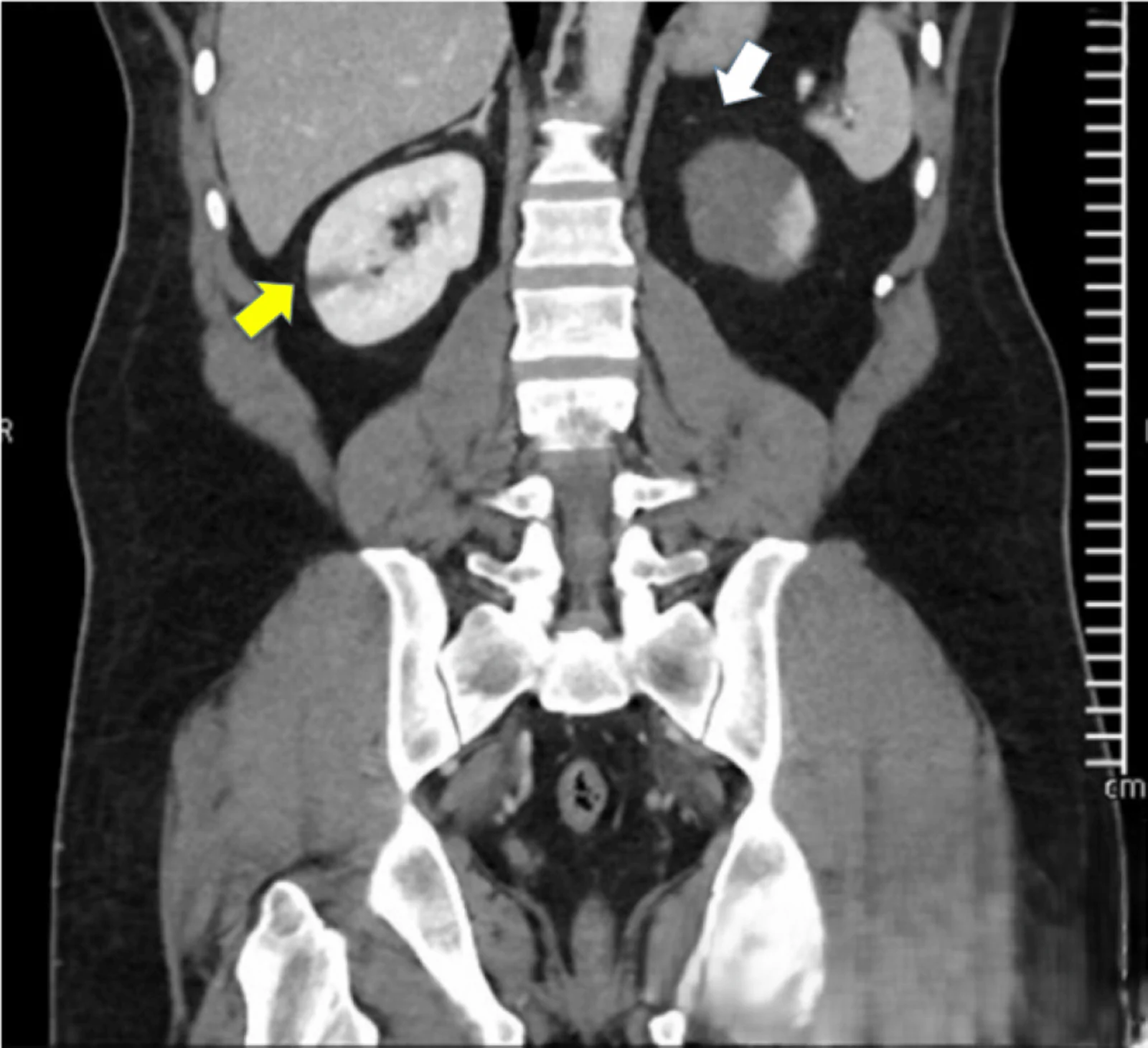
Contrast-enhanced computed tomography of the abdomen and pelvis revealed thrombosis of the left renal artery(white arrow) with complete infarction of the left kidney, along with a small segmental infarction in the posterior aspect of the mid-pole of the right kidney (yellow arrow)

A comprehensive evaluation for thromboembolic disorder was performed. Prothrombin time, activated partial thromboplastin time, and platelet count were within normal limits. The thrombophilia workup revealed reduced protein C activity (Table 1). He underwent further evaluation for systemic vasculitis, including an appropriate vasculitis workup, which was negative. As the patient had no clinical signs or symptoms suggestive of active tuberculosis, a specific evaluation for tuberculosis was not performed. The echocardiogram was normal, with no evidence of intracardiac thrombus or other potential cardiac source of embolism. The patient weighed 60 kg at the time of presentation and was initially treated with enoxaparin 60 mg twice daily as therapeutic anticoagulation. Rivaroxaban was initiated the following day, and the patient was subsequently discharged on rivaroxaban 20 mg once daily for continued anticoagulation. During hospitalization, the patient's flank pain and abdominal discomfort progressively improved, while serum creatinine changed from 1.5 mg/dl on admission to 1.1 mg/dL at discharge, with a corresponding estimated glomerular filtration rate (eGFR) value of 85 mL/min/1.73 m². Urine output remained adequate during the admission. The patient was discharged after six days of hospitalization in clinically stable condition. At the first outpatient assessment two weeks after discharge, he remained asymptomatic, and serum creatinine was 1.0 mg/dL with an eGFR of 90 mL/min/1.73 m².

**Table 1:** Laboratory investigation of acute kidney injury and hypercoagulability conditions LDH: lactate dehydrogenase; AST: aspartate aminotransferase; ANA: antinuclear antibody; PR3/MPO ANCA: proteinase 3 (PR3) and myeloperoxidase (MPO) anti-neutrophil cytoplasmic antibodies (ANCA)

Investigation	Normal value	Patient value	Result
LDH	100-250U/L	560U/L	abnormal
AST	0-32 U/L	34 U/L	normal
Antithrombin III	80-120%	126%	abnormal
Factor V Leiden	NA	Mutation absent	normal
Protein S	72-126%	99%	normal
Anticardiolipin Ab	Not applicable	Negative	normal
Anti-dsDNA Ab	Not applicable	Negative	normal
ANA	Not applicable	Negative	normal
PR3/MPO ANCA	Not applicable	Negative	normal
C3	90-180 mg/dl	125mg/dl	normal
C4	12-42 mg/dl	23mg/dl	normal
Protein C	70-140%	23%	reduced
Lupus anticoagulant	Not applicable	Negative	normal
Urinary sediment	Not applicable	leukocytes +, erythrocytes +	abnormal
24hr urinary protein	0-150mg	400mg	abnormal

## Discussion

Renal artery thrombosis is an uncommon cause of renal infarction but a potentially serious cause of acute kidney injury (AKI) and may be difficult to diagnose because its clinical presentation is nonspecific. It results from an abrupt interruption of renal arterial blood flow and may involve the main renal artery, segmental branches, or smaller intrarenal vessels. Renal infarction is most frequently associated with cardioembolic disease, particularly atrial fibrillation, although in situ renal artery thrombosis, aortic thromboembolism, renal artery dissection, trauma, vasculitis, and inherited or acquired hypercoagulable states are also recognized causes. Although renal artery thrombosis can occur in all age groups, it most commonly affects adults between 30 and 50 years of age. Patients usually present with the sudden onset of severe, persistent flank pain. It may be associated with fever, nausea, and vomiting. Hematuria and proteinuria are also frequently observed. The differential diagnosis of acute bilateral flank pain with AKI is broad.

Nephrolithiasis is one of the most common considerations, particularly when the pain is colicky; however, the absence of calculi or urinary tract obstruction on imaging should prompt reconsideration of the diagnosis. Acute pyelonephritis may produce flank pain, fever, pyuria, and renal dysfunction, but usually has supporting urinary and systemic inflammatory findings. Acute tubular injury, acute interstitial nephritis, rapidly progressive glomerulonephritis, renal vein thrombosis, renal artery dissection, atheroembolic renal disease, and bilateral obstructive uropathy should also be considered depending on the clinical context. Due to its nonspecific clinical presentation, the diagnosis is often delayed, which may result in a high risk of irreversible renal impairment. Furthermore, reperfusion therapies are less effective once prolonged ischemic injury has occurred [8]. Early recognition of renal infarction is essential, and initial evaluation should include renal function testing, urinalysis, lactate dehydrogenase (LDH), complete blood count, inflammatory markers, coagulation studies, and assessment for hematuria and proteinuria. Occlusion of a main renal artery can therefore produce extensive renal ischemia, while segmental arterial occlusion produces localized infarction. The severity and reversibility of renal injury depend on the extent of vascular occlusion, the duration of ischemia, the degree of residual perfusion, the presence of collateral circulation, and whether one or both kidneys are affected. The patient initially underwent a non-contrast CT scan to exclude nephrolithiasis; however, no renal calculi were identified. In view of the persistent flank pain, markedly elevated lactate dehydrogenase (LDH) levels, and AKI, the suspicion for renal infarction remained high. Consequently, a contrast-enhanced CT scan was performed, which revealed bilateral renal infarction with left renal artery thrombosis.

Ultrasound and non-contrast CT are the preferred initial imaging modalities for evaluating patients presenting with flank pain and hematuria, as they help exclude common conditions such as urolithiasis and pyelonephritis. Renal CT angiography is considered the gold standard for diagnosing renal infarction. Other imaging methods that can be used for further evaluation include magnetic resonance imaging (MRI) with gadolinium contrast and renal MR angiography. Following the diagnosis of renal infarction, further evaluation should include an electrocardiogram (ECG) to assess for atrial fibrillation as a potential embolic source. An echocardiogram should also be performed to evaluate for a cardioembolic etiology, particularly in patients with a history of cardiac disease or other risk factors for cardiac thromboembolism. Given the rarity of renal infarction, evidence-based treatment guidelines remain lacking [9]. Treatment strategies for acute renal infarction should be individualized based on the time elapsed since symptom onset and the precipitating cause. When endovascular intervention is considered, thrombectomy is typically performed as the initial approach, with thrombolysis reserved for selected cases. The recommended door-to-treatment interval is approximately 90-180 minutes [10]. In patients with delayed presentation, anticoagulation remains the cornerstone of management and is usually maintained for at least six months. The exact onset of renal infarction is often unclear, making treatment decisions more challenging. The outcome is influenced by the size of the clot, the duration of the blockage, and the extent of kidney damage. Most patients show improvement in renal function once renal perfusion is restored. The need for renal replacement therapy is uncommon, occurring in less than 10% of cases. The literature reports a limited number of cases of bilateral renal infarction and renal artery thrombosis in which renal function recovery was achieved with anticoagulation therapy. Similarly, our patient achieved significant renal function improvement with anticoagulation alone. Following initial treatment with low-molecular-weight heparin, the patient was discharged on rivaroxaban for at least six months, with subsequent follow-up arranged with the hematology clinic, including repeat protein C testing to reassess protein C activity. This case highlights renal artery thrombosis managed with enoxaparin followed by rivaroxaban, with subsequent improvement in renal function.

Tuberculosis (TB), caused by *Mycobacterium tuberculosis*, is an airborne infectious disease that primarily involves the respiratory system. Beyond pulmonary involvement, TB can exert systemic effects on the body. It has been shown to influence physiological processes, including the coagulation system, thereby contributing to a hypercoagulable state. Patients with active TB have an increased risk of developing venous thromboembolism (VTE). Studies show that people with TB have a higher risk of developing VTE, including deep vein thrombosis (DVT) and pulmonary embolism (PE). A meta-analysis by Danwang also reported an increased risk of VTE among individuals with TB [11]. In TB, inflammatory cells, cytokines, and other immune factors contribute to the formation of granulomas and can also disturb the body's normal balance and homeostasis [12]. These changes include increased blood-clotting activity, shown by abnormalities in prothrombin time (PT), activated partial thromboplastin time (aPTT), fibrinogen, and D-dimer levels. They also involve reduced levels of natural anticoagulants, such as antithrombin III, protein S, and protein C, along with decreased fibrinolysis. Together, these changes create a hypercoagulable state, increasing the risk of thrombosis. We present this case of bilateral renal infarction because renal artery thrombosis is a rare clinical entity, and bilateral renal artery infarction is exceptionally uncommon. In this patient, the diagnostic process was initially challenging because he presented primarily with sudden-onset flank pain and only mild renal dysfunction. The initial noncontract CT performed using a renal stone protocol did not identify an explanatory abnormality. However, the persistence of unexplained flank pain together with an elevated lactate dehydrogenase level and increased serum creatinine raised suspicion for a renal vascular process. This prompted contrast-enhanced CT, which demonstrated left renal arterial thrombosis with extensive renal infarction and a small contralateral segmental infarction. This sequence illustrates the importance of maintaining a high index of suspicion for renal infarction when the initial evaluation for more common causes of flank pain is unrevealing. The etiology of renal arterial thrombosis in this patient remains uncertain. Common mechanisms of renal infarction include cardiac or aortic embolism, in situ renal arterial thrombosis, renal artery dissection, trauma, and underlying coagulation disorders. In this case, there was no reported history of cardiovascular disease, and the clinical evaluation did not identify an obvious cardiac or systemic embolic source. The imaging findings and vascular surgical assessment therefore favored an arterial thrombotic process rather than an alternative cause. Nevertheless, the available investigations do not establish a single definitive etiologic mechanism. A markedly reduced protein C activity of 23% was identified during the acute thrombotic event. This finding is potentially relevant because protein C is an important endogenous anticoagulant, and impaired protein C activity can contribute to a prothrombotic state. However, the interpretation of this result requires considerable caution. Protein C activity may be reduced transiently during acute thrombosis and systemic illness and can also be affected by acquired conditions and anticoagulant therapy. In this patient, repeat testing after improvement under appropriate conditions was not performed. Consequently, hereditary protein C deficiency cannot be diagnosed with this single measurement. The patient's history of tuberculosis represents another potential consideration, but its significance is also uncertain. Active tuberculosis is associated with systemic inflammation and a prothrombotic state, with reported alterations in endothelial function, platelet activation, coagulation, and fibrinolysis. In the present case, however, there was no evidence of active tuberculosis at the time of renal arterial thrombosis. Therefore, it would be inappropriate to conclude that tuberculosis caused the renal arterial thrombosis. A previous history of tuberculosis may have represented a potential background thrombotic factor, but this remains speculative.

The principal limitation of this case is, therefore, the inability to determine a definitive etiology for the renal arterial thrombosis. In particular, protein C activity was not repeated after recovery, and the patient did not have documented evidence of active tuberculosis at the time of the event. Accordingly, the case should not be interpreted as proof of tuberculosis-associated renal arterial thrombosis or hereditary protein C deficiency. Rather, it highlights the diagnostic importance of considering renal vascular disease in patients with unexplained flank pain and elevated lactate dehydrogenase, while illustrating the challenges of determining causality when multiple potential thrombotic risk factors are present. To the best of our knowledge, bilateral renal infarction with renal artery thrombosis occurring in association with reduced protein C levels and recently treated tuberculosis is exceptionally rare.

## Conclusions

Renal arterial thrombosis is a rare but potentially devastating cause of acute kidney injury that requires a high index of suspicion, particularly in patients with unexplained flank pain and acute kidney injury. Early contrast-enhanced vascular imaging can facilitate timely diagnosis and guide management. Potential prothrombotic factors, including recently treated tuberculosis and reduced protein C activity, should be considered, although their causal role in this case could not be established.
